# Hay Fever

**Published:** 1877-10

**Authors:** 


### Hay Fever.

A gentleman who has made four journeys to Egypt and Palestine writes us as follows: "I have had occasion to try the Racquette Lake region for hay fever; have been there several times with good results. But Palestine or Egypt is much better. The good effect lasts seveaal years in my case. I consider that it is due to open-air life, under a tent, in a very dry climate, or on the desert." If this experience is not an exceptional one, it will tend still further to mystify our notions of the causation and treatment of this already sufficiently mysterious malady.

The last Report of the Hay Fever Association (F. B. Fay, Chelsea, Mass., Secretary), printed in June of this year, and just received, states that the nitrate of amyl was used last year by a few patients, with no little benefit. Inhalations of the amyl are taken directly from the bottle. The bromides are named, along with forty other prescriptions and drugs. The vapor of chloral hydrate has also been recently recommended; but no results have been published of its trial.—*Proceedings of the Medical Society of the County of Kings.*

